# Investigating students’ attitudes toward poverty and impoverished persons - A case study: Ho Chi Minh City Open University, data of Vietnam

**DOI:** 10.1016/j.dib.2021.107788

**Published:** 2022-01-02

**Authors:** Le Minh Tien

**Affiliations:** Faculty of Sociology-Social Work-Southeast Asian Studies, Ho Chi Minh City Open University, Ho Chi Minh City, Vietnam

**Keywords:** Social sciences, Social work, Students, Poverty, Attitudes, Vietnam

## Abstract

Efforts to understand the causes of poverty, how poverty is perceived have become important in the fight to mitigate poverty. In Vietnam, studies on the attitudes of poverty in specific populations, such as Vietnamese students, are rare. Thus, this dataset reports the results collected from 180 social work students and non-social work students of Ho Chi Minh City Open University through attitudes toward poverty and poor people in Vietnam. The Attitude toward Poverty Short Form 21-item scale, developed by Yun & Weaver, was used for the data collection. The survey results showed that when looking for causes of poverty, social work students and non-social work students put the most emphasis on structural factors of poverty. However, social work students, compared with non-social work students, consider personal deficiency and stigma more important. In future, this dataset can serve as a reference source for comparative studies on student’ attitudes toward poverty and impoverished persons and for social work education.

## Specifications Table

**Table:** 

Subject	Social Sciences
Specific subject area	social work, social work education, poverty, attitude toward poverty, higher education
Type of data	Raw data in excel file SPSS File
How data were acquired	Survey with questionnaire (included in Supplementary Materials)
Data format	Raw Independent sample t-test
Parameters for data collection	Participants who are full-time social work and non-social work students at Ho Chi Minh City Open University in Vietnam decided to take part in the survey voluntarily.
Description of data collection	Data were collected by convenience nonprobability sampling and based on survey questionnaire. The questionnaire was designed on the Attitude toward Poverty Short Form (ATP-SF) includes 21-item and the questionnaire was distributed to students in August, 2020. After obtaining permission from lecturers, the author administered questionnaires to students in their classrooms. Students were told that the study was to explore their perceptions of poverty, and were told that the study was voluntary and anonymous. The dataset includes 180 valid observations.
Data source location	Author’ survey, with sample of 180 students Institution: Ho Chi Minh City Open University City/Town/Region: Ho Chi Minh City Region: Asia Country: Vietnam Latitude and longitude (and GPS coordinates, if possible) for collected samples/data: 21.028511, 105.804817
Data accessibility	Repository name:The data sets are deposited on Mendeley Data and can be accessed via the following link: doi: 10.17632/bk4hh37rfr.2 Data identification number: Direct URL to data: https://data.mendeley.com/datasets/bk4hh37rfr/2

## Value of the Data

• The dataset covered information of social work and non-social work students’ attitudes on poverty and poor population. It's first survey of student's attitudes toward poverty in Vietnam.
• The dataset is useful for further comparing researches on perceptions of poverty among diverse countries because the data were collected in Vietnam, a country in Southeast Asia region.
• The dataset can be serve as a reference source for social work education and for improvement of the quality of social work teaching in other universities. It can also be useful for service users and social workers in practice.
• The dataset could provide a base for the development of an educational program to prepare social workers to care for the special needs of the poor population. The social work lecturers could use this dataset to understand student attitudes toward the poverty and/or the poor population when they are addressing value questions associated with poverty.

## 1 Data Description

The social work profession is considered as a profession that committed to help the poor and disadvantaged population because the social work is a profession rather than an ideology. Therefore, several studies have investigated attitudes toward poverty and poor population among students [2], [3], [4], [5], [6], [7], [8]. The dataset is a survey on Vietnamese social work (SW) and non-social work (NSW) students’ attitudes toward the poverty and the impoverished persons. To my knowledge, this paper is the first to measure the students’ attitudes toward poverty in Vietnam. This article is associated with a Excel format as supplementary material. The data file contains students' demographic characteristics and students’ agreements or disagreements with the “questionnaire” statements. The respondents evaluated agreements and disagreements using a 5-point Likert scale, where 1 – for “strongly agree” and 5 for “strongly disagree”.

The original questionnaire is provided in Vietnamese. The questionnaire includes 2 parts: the first section contains items collecting information about the respondent's characteristics, including gender, university year of studying, age, academic performance, ethnicity, student's family economic status (see Table 1). The second part consists of 21 items related to perceptions of respondents about poverty and/or impoverished persons including three dimensions: personal deficiency, stigma and structural perspective, developed by Yun & Weaver [1] (see Table 2). To complete the form, students spent about 15 min answering all questions. The results collected 180 valuable responses. An independent-samples *t*-test (one-tailed) was conducted to compare means between SW and NSW students (see Table 3). The raw data (Excel format, SPSS format) and questionnaire (MS Word) are available in the Supplementary Materials.

**Table 1:** Demographic characteristics of the sample.

Characteristics	N	%
*Gender*	*180*	*100.0*
Male	42	23.3
Female	138	76.7
*University year of studying*	*180*	*100.0*
Second year	105	58.3
Third year	75	41.7
*Fields of study*	*180*	*100.0*
Social work	86	47.8
Non-social work	94	52.2
*Academic performance*	*176*	*97.8*
Very good	17	9.4
Fair	112	62.2
Average	47	26.1
*Missing*	*4*	*2.2*
*Family economic status*	*180*	*100.0*
High income	60	33.3
Middle income	109	60.6
Low income	11	6.1
*Ethnicity*	*178*	*98.9*
Kinh (vietnamese)	173	96.1
Others	5	2.8
*Missing*	*2*	*1.1*
*Age*	*180*	*100.0*
Mean	20.5	
Std. Deviation	0.95

**Table 2:** Descriptive results of participants’ responses.

Variables		N	Min	Max	Mean	Std. Deviation
*Personal deficency (PD) (Cronbach's Alpha = 0.698)*						

PD1	Poor people are different from the rest of society	178*	1	5	3.74	0.90
PD2	Poor people are dishonest	180	2	5	4.38	0.73
PD3	Most poor people are dirty	178*	2	5	4.57	0.67
PD4	Poor people act differently	179⁎⁎	1	5	4.15	0.84
PD5	Children raised on welfare will never amount to anything	179⁎⁎	3	5	4.60	0.55
PD6	I believe poor people have a different set of values than do other people	179⁎⁎	1	5	2.92	1.17
PD7	Poor people generally have lower intelligence than nonpoor people	180	2	5	4.34	0.78

*Stigma (STg) (Cronbach's Alpha = 0.652)*						

STg1	There is a lot of fraud among welfare recipients	180	1	5	2.53	0.86
STg2	Some "poor" people live better than I do, considering all their benefits	180	1	5	3.12	0.87
STg3	Poor people think they deserve to be supported	180	1	5	2.87	0.86
STg4	Welfare mothers have babies to get more money	180	1	5	3.26	1.03
STg5	An able-bodied person collecting welfare is ripping off the system	180	1	5	3.07	1.08
STg6	Unemployed poor people could find jobs if they tried harder.	180	1	5	2.23	1.00
STg7	Welfare makes people lazy	180	1	5	2.92	0.98
STg8	Benefits for poor people consume a major part of the state budget	180	1	5	3.03	0.77

*Structural perspective (SP) (Cronbach's Alpha = 0.586)*						

SP1	People are poor due to circumstances beyond their control	179⁎⁎	1	5	2.71	1.07
SP2	I would support a program that resulted in higher taxes to support social programs for poor people	180	1	5	3.34	0.99
SP3	If I were poor, I would accept welfare benefits	180	1	5	2.54	0.88
SP4	People who are poor should not be blamed for their misfortune	179⁎⁎	1	5	1.92	0.87
SP5	Society has the responsibility to help poor people	180	1	5	2.45	0.93
SP6	Poor people are discriminated against	180	1	5	2.31	0.89

*Note*:

⁎ Missing: 2 cases;

⁎⁎ Missing: 1 case.

**Table 3:** Independent-samples *t*-test.

Dimensions	Group of students	N	Mean	t	df	*P*-value
Personal deficiency	SW NSW	86 94	3.97 4.23	−3.709	178	0.000
Stigma	SW NSW	86 94	2.71 3.04	−4.639	178	0.000
Structural perspective	SW NSW	86 94	2.52 2.58	−0.776	178	0.439

*Note*: SW = Social work; NSW = Non-social work.

One hundred eighty undergraduate students participated in the survey. Among them, 86 were social work students, the other 94 students were from law (*n* = 34), foreign language (*n* = 30) and economics (*n* = 30). Gender distribution included 42 males (23.3%) and 138 females (76.7%). The mean age of the entire sample was 20.5 years old (SD=0.95). The demographic characteristics of the sample are summarized in Table 1.

As seen from Table 1, the majority of students who completed the survey were females because the number of female students of Ho Chi Minh City Open University was more than seventy percent. The university year of study distribution included 105 (58.3%) second-year students and 75 (41.7%) third-year students. The mean age of the entire sample was 20.5 years old (SD = 0.95).

Table 2 presents the mean and standard deviation of the ATP-SF 21 items and the Cronbach's alpha coefficient of the three dimensions of poverty. According to Taber (2018) [9], a reliability value of Cronbach's alpha between 0.58 and 0.97 qualifies for satisfactory reliability of the scale measured, while a value greater than 0.70 shows relatively high internal consistency . Thus, the three subscales of the questionnaire had satisfactory reliability. Specifically, the Cronbach's alpha coefficient for *the personal deficiency* factor was 0.698, the Cronbach's alpha coefficient for *the stigma* factor was 0.652 and the Cronbach's alpha coefficient for *the structural perspective* factor was 0.586. Thus, compared to the Yun & Weaver study (2010), the Cronbach's alpha coefficients of this study were lower.

As seen from Table 3, when looking for causes of poverty, SW students and NSW students place the most emphasis on structural factors of poverty. However, SW students, compared with NSW students, consider personal deficiency and stigma more important.

## 2 Experimental Design, Materials and Methods

The data were collected in August 2020, the first semester of the academic year 2020–2021. Participants who are full-time students at Ho Chi Minh City Open University, Ho Chi Minh City, Vietnam decided to take part in the survey voluntarily. The survey were conducted by using convenience nonprobability sampling and based on survey questionnaire.

**Table 4:** The coding of demographic variables were performed as:

Variables	Code	Value
SW	1	Social work student
	2	Non-social work student

MAJOR	1	Social work
	2	Law
	3	Foreign language
	4	Economics

YEAR	1	Second year
	2	Third year

SEX	1	Male
	2	Female

ETHNIC	1	Kinh/Vietnamese
	2	Hoa/Chinese
	3	Khác/Others

ECON	1	High income
	2	Middle income
	3	Low income

ACADEMIC	1	Very good
	2	Fair
	3	Average
	4	Poor

The questionnaire items have been used from previous research, developed by Yun and Weaver [1]. The Yun & Weaver’ Attitude toward Poverty Short Form (ATP-SF) includes 21-item. The 21-item instrument used to measure student attitudes toward poverty on three dimensions: *personal deficiency* (7 items), *stigma* (8 items), and *structural perspective* (6 items) of poverty. The items are scored on a 5-point Likert scale such as 1 = strongly agree, 2 = agree, 3 = neutral, 4 = disagree, 5 = strongly disagree. Higher scores denote strong negative attitudes toward three dimensions of poverty, and lower scores reflect more positive attitudes toward three dimensions of poverty. Most likely, this was the first application of ATP-SF in the Vietnamese context.

However, as the participants are Vietnamese students, therefore, the items were first translated into Vietnamese from the original English version. Some words have been modified to be more suitable to the Vietnamese context (For example: “federal budget” is replaced by “state budget”). After obtaining permission from lecturers, the author administered questionnaires to students in their classrooms. Students were told that the study was to explore their perceptions of poverty, and were told that the study was voluntary and anonymous, respondents' names were not included in the data to maintain privacy. The coding were showed in Table 4.

The software of IBM Statistical Package for Social Sciences (SPSS) 20 was used in the data analysis process. Specifically, independent sample t-test was performed to compare two means which obtained from two groups of students with low different educational programs: social work and non-social work.

## Ethics Statement

The author kept to all ethical concerns during the data gathering process. The author ensured that all respondents’ information is used for research purposes and is absolutely confidential. Research has been conducted in an environment that does not require ethical approval for survey studies.

## CRediT Author Statement

**Le Minh Tien:** I did all the research work. No other scientific contributions.

## Declaration of Competing Interest

The author declares that there are no known competing financial interests or personal relationships that have or could be perceived to have influenced the work reported in this article.

## Appendix Supplementary materials

Image, application 1

Image, application 2
