# Supplementary material for: Investigating students’ attitudes toward poverty and impoverished persons - A case study: Ho Chi Minh City Open University, data of Vietnam
Source: Data Brief. 2022 Jan 2;40:107788. doi: 10.1016/j.dib.2021.107788 (PMC8741469; doi:10.1016/j.dib.2021.107788)
Supplement: Supplementary file 2 [file mmc2.docx]

**QUESTIONNAIRE**

*Attitudes Toward Poverty and Impoverished Persons*

Dear Students,

The purpose of this survey is to understand your attitudes toward the Poverty and the Impoverished Persons. Your participation and feedback will provide information that will enable OU to improve the quality of its education. I assure you that all the information included in this survey will be strictly confidential and will be used only for research purpose, future planning, and for developing the OU educational social work programs.

This questionnaire is in Vietnamese and it takes you around 15 munites to complete.

If you have questions, please contact:

Le Minh Tien, email: tien.lm@ou.edu.vn

Thank you so much for your cooperation.

***Part I. Demographic Information***

**1. Bạn hiện là sinh viên Khoa nào sau đây (Your specialization)?**

- Social work _1_

- Law _2_

- Foreign Languages _3_

- Economics _4_

**2. Bạn hiện là sinh viên năm thứ mấy (Year of study)?**

- Second year _1_

- Third year _2_

**3. Giới tính của bạn (Your gender)?**

- Male _1_

- Female _2_

**4. Tuổi của bạn (Your age)?**..........

**5. Bạn là người dân tộc nào (Your ethnicity)?**

- Kinh _1_

- Hoa/Chinese _2_

- Khác/others _3_

**6. Kinh tế gia đình của bạn (Your family’ income)?**

- High income _1_

- Middle income _2_

- Low income _3_

**7. Xin vui lòng cho biết xếp loại học tập của bạn trong học kỳ vừa qua (Your achievement last year)?**

- Very good _1_

- Fair _2_

- Average _3_

- Poor _4_

***Part II. Attitude toward the Poverty and the Impoverished Persons***

**Bạn vui lòng cho biết ý kiến của mình đối với từng nhận định theo thang đo sau** (Please, express honestly your agreement or disagreement with the following statement)

| **Phát biểu/statement** | (1)  Strongly Agree | (2)  Agree | (3)  Neutral | (4)  Disagree | (5)  Strongly Disagree |
| --- | --- | --- | --- | --- | --- |
| 1. Người nghèo khác với phần còn lại trong xã hội  Poor people are different from the rest of society |  |  |  |  |  |
| 2. Người nghèo không trung thực  Poor people are dishonest |  |  |  |  |  |
| 3. Hầu hết người nghèo đều dơ bẩn  Most poor people are dirty |  |  |  |  |  |
| 4. Người nghèo có ửng xử không giống mọi người  Poor people act differently |  |  |  |  |  |
| 5. Trẻ em được nuôi dưỡng nhờ trợ cấp xã hội sẽ không bao giờ đạt được thành công gì cả  Children raised on welfare will never amount to anything |  |  |  |  |  |
| 6. Tôi tin rằng người nghèo có một hệ giá trị khác với mọi người  I believe poor people have a different set of values than do other people |  |  |  |  |  |
| 7. Nói chung, người nghèo kém thông minh hơn người không nghèo  Poor people generally have lower intelligence than nonpoor people |  |  |  |  |  |
| 8. Có nhiều sự gian lận nơi những người nhận trợ cấp /phúc lợi xã hội  There is a lot of fraud among welfare recipients |  |  |  |  |  |
| 9. Một số người nghèo có cuộc sống tốt hơn tôi khi xem xét toàn bộ các lợi ích mà họ được hưởng  Some "poor" people live better than I do, considering all their benefits |  |  |  |  |  |
| 10. Người nghèo nghĩ rằng họ xứng đáng được hỗ trợ/trợ cấp  Poor people think they deserve to be supported |  |  |  |  |  |
| 11. Những bà mẹ sống nhờ trợ cấp sinh con để hưởng nhiều trợ cấp hơn  Welfare mothers have babies to get more money |  |  |  |  |  |
| 12. Người khỏe mạnh hưởng trợ cấp xã hội làm hủy hoại hệ thống phúc lợi  An able-bodied person collecting welfare is ripping off the system |  |  |  |  |  |
| 13. Những người nghèo thất nghiệp có thể kiếm được việc làm nếu họ cố gắng hơn  Unemployed poor people could find jobs if they tried harder. |  |  |  |  |  |
| 14. Trợ cấp/phúc lợi xã hội làm cho con người ta trở nên lười biếng  Welfare makes people lazy |  |  |  |  |  |
| 15. Lợi ích dành cho người nghèo chiếm phần đáng kể ngân sách quốc gia  Benefits for poor people consume a major part of the state budget |  |  |  |  |  |
| 16. Người ta nghèo là do những hoàn cảnh (thiên tai, dịch bệnh…) nằm ngoài sự kiểm soát của họ  People are poor due to circumstances beyond their control |  |  |  |  |  |
| 17. Tôi sẽ ủng hộ việc đánh thuế cao hơn để có tiền cho các chương trình xã hội trợ giúp người nghèo  I would support a program that resulted in higher taxes to support social programs for poor people |  |  |  |  |  |
| 18. Nếu tôi nghèo, tôi sẵn sàng nhận trợ cấp xã hội dành cho người nghèo  If I were poor, I would accept welfare benefits |  |  |  |  |  |
| 19. Không nên đỗ lỗi cho người nghèo  People who are poor should not be blamed for their misfortune |  |  |  |  |  |
| 20. Xã hội có trách nhiệm giúp đỡ người nghèo  Society has the responsibility to help poor people |  |  |  |  |  |
| 21. Người nghèo bị phân biệt đối xử  Poor people are discriminated against |  |  |  |  |  |

THANK YOU!

XIN CẢM ƠN BẠN ĐÃ THAM GIA
